# Meta-analysis of serological biomarkers at hospital admission for the likelihood of developing delirium during hospitalization

**DOI:** 10.3389/fneur.2023.1179243

**Published:** 2023-06-09

**Authors:** Thiago Bassi, Elizabeth Rohrs, Michelle Nicholas, Steven Reynolds

**Affiliations:** ^1^Lungpacer Medical USA Inc., Exton, PA, United States; ^2^Advancing Innovation in Medicine Institute, New Westminster, BC, Canada; ^3^Biomedical, Physiology and Kinesiology Department, Simon Fraser University, Burnaby, BC, Canada

**Keywords:** inflammation, hypothalamic-pituitary axis dysfunction, delirium, biomarkers, cholinergic burden

## Abstract

**Importance:**

Identifying biomarkers that, at hospital admission, predict subsequent delirium will help to focus our clinical efforts on prevention and management.

**Objective:**

The study aimed to investigate biomarkers at hospital admission that may be associated with delirium during hospitalization.

**Data sources:**

A librarian at the Fraser Health Authority Health Sciences Library performed searches from 28 June 2021 to 9 July 2021, using the following sources: Medline, EMBASE, Cochrane Database of Systematic Reviews, Cochrane Central Register of Controlled Trials, Cochrane Methodology Register, and the Database of Abstracts of Reviews and Effects.

**Study selection:**

The inclusion criteria were articles in English that investigated the link between serum concentration of biomarkers at hospital admission and delirium during hospitalization. Exclusion criteria were single case reports, case series, comments, editorials, letters to the editor, articles that were not relevant to the review objective, and articles concerning pediatrics. After excluding duplicates, 55 studies were included.

**Data extraction and synthesis:**

This meta-analysis followed the Preferred Reporting Items for Systematic Review and Meta-Analysis (PRISMA) protocol. Independent extraction, with the consensus of multiple reviewers, was used to determine the final studies included. The weight and heterogeneity of the manuscripts were calculated using inverse covariance with a random-effects model.

**Main outcome(s) and measure(s):**

Differences in mean serum concentration of biomarkers at hospital admission between patients who did and did not develop delirium during hospitalization.

**Results:**

Our search found evidence that patients who developed delirium during hospitalization had, at hospital admission, significantly greater concentrations of certain inflammatory biomarkers and one blood–brain barrier leakage marker than patients who did not develop delirium during hospitalization (differences in the mean: cortisol: 3.36 ng/ml, *p* < 0.0001; CRP: 41.39 mg/L, *p* < 0.00001; IL-6: 24.05 pg/ml, *p* < 0.00001; S100β 0.07 ng/ml, *p* < 0.00001). These differences were independent of other confounding variables such as the patient's severity of illness. A significantly lower serum concentration, at hospital admission, of acetylcholinesterase (difference in the means −0.86 U/ml, *p* = 0.004) was also associated with an increased vulnerability to developing delirium during hospitalization.

**Conclusion and relevance:**

Our meta-analysis supports the hypothesis that patients with hypothalamic-pituitary axis dysfunction, increased blood–brain barrier permeability, and chronic overload of the cholinergic system, at hospital admission, are more vulnerable to developing delirium during hospitalization.

## Introduction

Delirium is characterized by acute and transient changes in cognitive function, especially in attention (1, 2). Patients who develop delirium during hospitalization have a 3-fold higher 1-year mortality and an approximately 20-day greater length of stay than patients who do not develop delirium during hospitalization (3, 4). Although delirium is common during hospitalization, having an incidence range of 10–60%, its etiology is still not well understood (5, 6). Direct brain insults, chronic overactivation of the immunologic system, and overload of the cholinergic system have been proposed as causes of increased individual susceptibility to delirium during hospitalization (1).

Many factors may trigger delirium during hospitalization (1). It has been hypothesized that an acute overactivation of the immunologic system might affect neuronal synapses, leading to acute cognitive impairment and/or delirium (1). Patient susceptibility to developing delirium is therefore attributed, in part, to how the immunologic system responds to inflammation and stress (1, 7). Chronic inflammation has been shown to prime microglia activity and to increase blood–brain barrier (BBB) permeability, increasing the likelihood of overactivation of the immunologic system in the brain during acute inflammation and stress (1, 7). An inappropriate immunologic response to inflammation may result in an acute neurotransmitter imbalance, potentially manifesting as delirium (1, 7). Hypothalamic-pituitary axis (HPA) dysfunction has also been associated with chronic inflammation and inappropriate immunologic response during inflammation and stress (1, 7). HPA dysfunction is also linked to neurotransmitter imbalance and has been associated with decreased activity in the prefrontal cortex, resulting in disturbance of attention, memory, and executive functions (1, 7). While any one of these mechanisms might trigger delirium, all of them can coexist in a single patient (1). Many studies have tried to demonstrate an association between delirium and the presence of biomarkers in the serum (8–10). These biomarkers reflect different potential precipitants of delirium and may not only predict delirium but also give clues as to the likely mechanism.

Although the risk factors for delirium have been well documented, few studies have focused on the pathophysiology of delirium. We conducted a systematic review and meta-analysis on the association of serological biomarkers at hospital admission with the development of delirium during hospitalization. The primary objective was to summarize the current findings in the literature regarding serum concentration of biomarkers at hospital admission as predictors of the development of delirium during hospitalization. The second objective was to identify possible mechanisms for delirium. The third objective was to identify gaps in the literature.

## Methodology

**Diagram 1:**
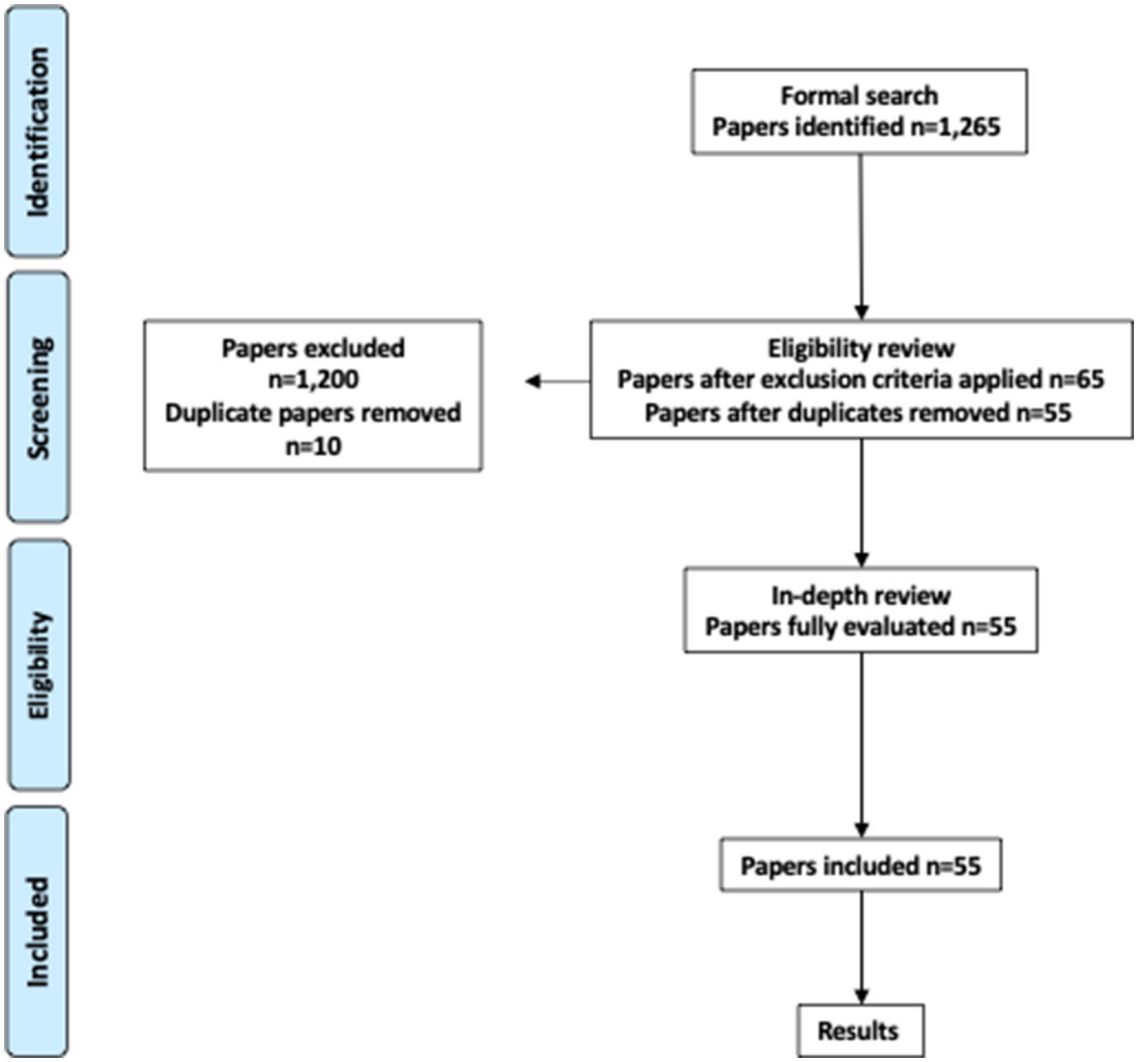
Systematic review process and results following PRISMA protocol.

This systematic review and meta-analysis followed the Preferred Reporting Items for Systematic Review and Meta-Analysis (PRISMA) protocol (Diagram 1). A librarian at the Health Sciences Library at Fraser Health Authority, Royal Columbian Hospital (New Westminster, Canada), performed the searches. Searches were conducted using the following sources: Medline (1946-present), EMBASE (1974-present), Cochrane Database of Systematic Reviews, Cochrane Central Register of Controlled Trials, Cochrane Methodology Register, and the Database of Abstracts of Reviews and Effects (DARE).

We developed search strategies based on the search interface to ensure an appropriate balance between search sensitivity and specificity. Articles were limited to published prospective and retrospective studies. Keyword, adjacency, wildcard, and subject-heading searching were employed in all search strategies to maximize the sensitivity of the search, while publication limits, specific clinical terms, and their variants were used to increase the specificity of the search results. Inclusion criteria were articles in English that investigated links between serum concentration of biomarkers at hospital admission and delirium during hospitalization. Exclusion criteria were single case reports, case series, comments, editorials, letters to the editor, articles that were not relevant to the review objective, and articles concerning pediatrics.

Formal searches for articles were conducted, using pre-established keywords, from 28 June 2021 to 9 July 2021 (Supplementary Table S1). A pre-screening review was conducted by the first author (TB) to eliminate articles that did not satisfy the inclusion criteria. Articles not eliminated during the pre-screening review then underwent an in-depth review. Each included manuscript was fully and independently evaluated by two out of the three reviewers (TB, ER, and MN). Articles were divided into three groups with a similar number of articles. Each group of articles was reviewed by one of the three unique pairings of reviewers, resulting in each reviewer assessing the risk of bias and quality of approximately two-thirds of the articles. The revised National Heart, Lung, and Blood Institute score system was used to assess the risk of bias. Covidence systematic review software (Veritas Health Innovation, Melbourne, Australia) was used to assess the quality of the articles. In the event of significant score discrepancies between the first two reviewers, the third reviewer independently evaluated the study and served as the adjudicator.

The reviewers independently identified the biomarkers that were investigated in each study. All types of biomarkers regardless of their biochemical specificity and the methodology applied to measure them were included in the study. However, only biomarkers that appeared in four or more studies were included in the meta-analysis. Studies that investigated surgical and non-surgical populations were included in the study. Differences between the means in serum concentration at hospital admission between patients who did and did not develop delirium during hospitalization were our primary outcome for the meta-analysis. Only the serum concentration at hospital admission was used to conduct the meta-analysis although studies reported serum concentrations at other timelines such as post-surgery and on day 1 or day 2 after hospital admission, for example. The weights and heterogeneity of the manuscripts were calculated using inverse covariance with a random-effects model. A *p*-value of < 0.1 for the chi-square test was considered to be statistically significant. Heterogeneity was evaluated using the Higgins metric (I^2^), where I^2^ > 75% was considered as having significant heterogeneity, I^2^ of 40–74% was considered moderate heterogeneity, and I^2^ < 39% was considered no heterogeneity. If high heterogeneity was found, a subgroup analysis was performed to investigate the cause of high heterogeneity. All statistical analyses were performed using the Review Manager software [RevMan, Version 5.4.1, The Cochrane Collaboration, (11)].

## Results

The formal searches identified 1, 265 manuscripts, of which 65 were selected after applying the inclusion and exclusion criteria. Fifty-five manuscripts were included as part of the systematic review after excluding duplicates (Table 1). All studies selected were observational/cohort, except one study that was interventional. Our assessment showed that all studies had a low risk of selection bias. The risk of performance bias (inadvertently introducing differences between groups) was assessed as high in 55%, unclear in 12%, and low in 33% of the studies. The risk of detection bias (difference in how to measure outcomes between groups) was assessed as high in 25%, and low in 75% of the studies. The risk of attribution bias (the tendency to favor one group as a result of individual beliefs) was assessed as low for all studies. The risk of reporting bias was high in 36% and low in 64% of the studies. For the 55 studies selected, quality assessment scored 15 (27%) as “good,” 36 (65%) as “fair,” and 4 (7%) as “poor.” The most common population studied was post-orthopedic surgical patients (19 publications), followed by cardiovascular surgical patients (14), ICU patients (12), and general-ward patients (10).

**Table 1:** Table showing the papers included in the meta-analysis.

**Authors**	**Year**	**Title**	**Type of study**	**Population studied**	**Number of subjects**	**Quality score**	**Delirium assessment tool**	**Cognitive assessment tool**	**Main Biomarkers studied**
Adam et al. (12)	2020	Cholinesterase alterations in delirium after cardiosurgery: A German monocentric prospective study	Observational study	PO orthopedic surgery	114	Fair	Confusion assessment method-ICU	Not assessed	Acetylcholinesterase (AChE)
Adamis et al. (13)	2007	APOE and cytokines as biological markers for recovery of prevalent delirium in elderly medical inpatients	Observational study	General ward patients	164	Fair	Confusion assessment method	Mini-mental state examination	IL-1β, IL-1ra, IL-6, TNF-a, IGF-1, IFN, LIF, CRP, APOE genotype
Alexander et al. (14)	2014	Interleukin 6 and apolipoprotein e as predictors of acute brain dysfunction and survival in critical care patients	Observational study	ICU patients	53	Fair	Confusion assessment method-ICU	Assessed retrospectively via past medical history	APOE, IL-6, IL-8, IL-10
Anderson et al. (15)	2016	Admission plasma levels of the neuronal injury marker neuron-specific enolase are associated with mortality and delirium in sepsis	Observational study	General ward patients	124	Fair	Confusion assessment method-ICU	Not assessed	NSE
Ballweg et al. (16)	2021	Association between plasma tau and postoperative delirium incidence and severity: a prospective observational study	Observational study	General ward patients	114	Fair	Confusion assessment method-ICU	Not assessed	GFAP, IL-1β, IL-6, IL-8, IL-10, NfL, TNFα, Tau,
Beloosesky et al. (17)	2007	Cytokines and C-Reactive Protein Production in Hip-Fracture-Operated Elderly Patients	Observational study	PO orthopedic surgery	38	Fair	Confusion assessment method	Mini-mental state examination	CRP, IL-1ra, IL-1b, IL-6, IL-8, IL-10, TNFα
Casey et al. (18)	2020	Postoperative delirium is associated with increased plasma neurofilament light	Observational study	PO orthopedic surgery	108	Fair	Confusion assessment method-ICU	Not assessed	IL-1β, IL-1ra, IL-2, IL-4, IL-6, IL-8, IL-10, IL-12, MCP, NfL, TNFα
Cereghetti et al. (19)	2017	Independent Predictors of the Duration and Overall Burden of Postoperative Delirium After Cardiac Surgery in Adults: An Observational Cohort Study	Observational study	PO cardiac surgery	618	Good	Intensive care delirium screening checklist score	Not assessed	CRP
Cerejeira et al. (20)	2012	The cholinergic system and inflammation: Common pathways in delirium pathophysiology	Observational study	PO orthopedic surgery	101	Fair	Confusion assessment method	Mini-mental state examination	AchE and BuChE
Chen et al. (21)	2019	Change in serum level of interleukin 6 and delirium after coronary artery bypass graft	Observational study	PO cardiac surgery	266	Good	Confusion assessment method	Not assessed	IL-6
Colkesen et al. (22)	2013	Relation of serum cortisol to delirium occurring after acute coronary syndromes	Observational study	PO cardiac surgery	52	Fair	Delirium rating scale	Not assessed	Cortisol
De Rooij et al. (23)	2007	Cytokines and acute phase response in delirium	Observational study	General ward patients	185	Poor	Confusion assessment method-ICU	Mini-Mental State Examination and Informant Questionnaire on Cognitive Decline	CRP, IL-1 β, IL-6, IL-8, IL-10, TNFα
Egberts et al. (24)	2019	Differences in potential biomarkers of delirium between acutely ill medical and elective cardiac surgery patients	Observational study	PO orthopedic surgery	211	Fair	Delirium observation screening scale	Mini-mental state examination	Neopterin, HVA, Trypophan/LNAA
Erikson et al. (25)	2019	Elevated serum S-100β in patients with septic shock is associated with delirium	Observational study	General ward patients	22	Fair	Confusion assessment method-ICU	Not assessed	IL-6 and S100B
Fong et al. (26)	2020	Association of Plasma Neurofilament Light with Postoperative Delirium	Observational study	PO orthopedic surgery	162	Good	Confusion assessment method-ICU	General Cognitive Performance scores	NfL, GFAP, UCH-L1
Gao et al. (27)	2018	Transcutaneous electrical acupoint stimulation for prevention of postoperative delirium in geriatric patients with silent lacunar infarction: A preliminary study	Interventional study	PO orthopedic surgery	64	Poor	Confusion assessment method-ICU	Mini-mental state examination	S100β
Girard et al. (10)	2012	Associations of markers of inflammation and coagulation with delirium during critical illness	Observational study	ICU patients	138	Good	Confusion assessment method-ICU	Not assessed	CRP, MMP-9, MPO, NGAL, STNFR1, D-Dimer, Protein C, PAI-1, VWF
Grandi et al. (28)	2011	Brain-derived neurotrophic factor and neuron-specific enolase, but not S100β, levels are associated to the occurrence of delirium in intensive care unit patients	Observational study	ICU patients	60	Fair	Confusion assessment method-ICU	Not assessed	BDNF, NSE, S100B
Jackson et al. (29)	2017	Acetylcholinesterase Activity Measurement and Clinical Features of Delirium	Observational study	General ward patients	110	Fair	Confusion assessment method-ICU and abbreviated mental test score	Not assessed	AChE
Jones et al. (30)	2012	Analysis of neuron-specific enolase and S100B as biomarkers of cognitive decline following surgery in older people	Observational study	PO orthopedic surgery	68	Fair	Confusion assessment method-ICU	Mini-mental state examination and cognitive evaluation included global cognitive function	S100B and NSE
Jorge-Ripper et al. (31)	2017	Prognostic value of acute delirium recovery in older adults	Observational study	General ward patients	144	Fair	Confusion assessment method-ICU	Pfeiffer index	S100B, IL-6, IL-10, IFNβ, TNFα
Kazmierski et al. (32)	2013	Cortisol levels and neuropsychiatric diagnosis as markers of postoperative delirium: A prospective cohort study	Observational study	PO cardiac surgery	113	Fair	Confusion assessment method-ICU	Montreal Cognitive assessment and the trail making test	Cortisol, IL-2
Kazmierski et al. (33)	2014	Mild cognitive impairment with associated inflammatory and cortisol alterations as independent risk factor for postoperative delirium	Observational study	PO cardiac surgery	113	Fair	Confusion assessment method-ICU	Montreal cognitive assessment and the trail making test	Cortisol, IL-2 and TNFα
Khan et al. (34)	2013	S100 calcium binding protein B as a biomarker of delirium duration in the intensive care unit - An exploratory analysis	Observational study	ICU patients	63	Fair	Confusion assessment method-ICU	Not assessed	S100B
Knaak et al. (35)	2019	C-reactive protein for risk prediction of post-operative delirium and post-operative neurocognitive disorder	Observational study	PO orthopedic surgery	314	Fair	Confusion assessment method-ICU	Mini-mental state examination	CRP
Li et al. (36)	2019	Relation of postoperative serum S100A12 levels to delirium and cognitive dysfunction occurring after hip fracture surgery in elderly patients	Observational study	PO orthopedic surgery	186	Fair	Confusion assessment method-ICU	Mini-mental state examination	S100B
Liu et al. (37)	2013	High serum interleukin-6 level is associated with increased risk of delirium in elderly patients after noncardiac surgery: A prospective cohort study	Observational study	PO orthopedic surgery	338	Fair	Confusion assessment method-ICU	Mini-mental state examination	IL-6
Lu et al. (38)	2020	Usefulness of postoperative serum translocator protein as a predictive marker for delirium after breast cancer surgery in elderly women	Observational study	General ward patients	152	Fair	Confusion assessment method-ICU	Not assessed	CRP, TP
Lv et al. (39)	2021	Plasma interleukin-6 is a potential predictive biomarker for postoperative delirium among acute type a aortic dissection patients treated with open surgical repair	Observational study	PO cardiac surgery	221	Fair	Confusion assessment method-ICU	Not assessed	IL-6
Ma et al. (40)	2020	Age, preoperative higher serum cortisol levels, and lower serum acetylcholine levels predict delirium after percutaneous coronary intervention in acute coronary syndrome patients accompanied with renal dysfunction	Observational study	PO cardiac surgery	119	Good	Confusion assessment method-ICU	Not assessed	Cortisol and AchE
McGrane et al. (41)	2011	Procalcitonin and C-reactive protein levels at admission as predictors of duration of acute brain dysfunction in critically ill patients	Observational study	ICU patients	87	Fair	Confusion assessment method-ICU	Informant Questionnaire on Cognitive Decline in the Elderly	CRP, PCT
McKay et al. (42)	2021	Preliminary Study of Serum Biomarkers Associated With Delirium After Major Cardiac Surgery	Observational study	PO cardiac surgery	24	Good	Confusion assessment method	3d cognitive assessment method and telephone-montreal cognitive assessment	IL-6
McNeil et al. (8)	2019	Plasma biomarkers of inflammation, coagulation, and brain injury as predictors of delirium duration in older hospitalized patients	Observational study	General ward patients	156	Fair	Confusion assessment method-ICU	Informant Questionnaire on Cognitive Decline in the Elderly	IL-6, IL-8, sTNFRI, S100B
Mu et al. (43)	2010	High serum cortisol level is associated with increased risk of delirium after coronary artery bypass graft surgery: A prospective cohort study	Observational study	PO cardiac surgery	243	Good	Confusion assessment method-ICU	Not assessed	Cortisol
Müller et al. (44)	2019	Relevance of peripheral cholinesterase activity on postoperative delirium in adult surgical patients (CESARO): A prospective observational cohort study	Observational study	PO orthopedic surgery	650	Fair	Nursing delirium screening scale	Not assessed	AChE, butyrylcholinesterase (BuChE)
Nguyen et al. (45)	2014	Cortisol is an associated-risk factor of brain dysfunction in patients with severe sepsis and septic shock	Observational study	ICU patients	128	Good	Confusion assessment method-ICU	Not assessed	Cortisol, S100B
Osse et al. (46)	2012	High preoperative plasma neopterin predicts delirium after cardiac surgery in older adults	Observational study	PO cardiac surgery	125	Fair	Confusion assessment method-ICU	Mini-mental state examination	CRP, Neopterin, HVA, BH4
Peng et al. (47)	2019	Preoperative C-Reactive Protein/Albumin Ratio, a Risk Factor for Postoperative Delirium in Elderly Patients After Total Joint Arthroplasty	Observational study	PO orthopedic surgery	272	Good	Confusion assessment method-ICU	Mini-mental state examination	CRP, albumin ratio, IL-6
Plaschke et al. (48)	2010	Early postoperative delirium after open-heart cardiac surgery is associated with decreased bispectral EEG and increased cortisol and interleukin 6	Observational study	PO cardiac surgery	114	Fair	Confusion assessment method-ICU	Not assessed	Cortisol, IL-6
Ren et al. (49)	2020	Elevated Level of Serum C-reactive Protein Predicts Postoperative Delirium among Patients Receiving Cervical or Lumbar Surgery	Observational study	PO orthopedic surgery	206	Fair	Confusion Assessment Method and Memorial delirium assessment scale	Not assessed	CRP
Ritchie et al. (50)	2014	The association between C-reactive protein and delirium in 710 acute elderly hospital admissions	Observational study	General ward patients	710	Poor	Confusion assessment method-ICU	Not assessed	CRP
Ritter et al. (51)	2014	Inflammation biomarkers and delirium in critically ill patients	Observational study	ICU patients	78	Good	Confusion assessment method-ICU	Not assessed	IL-1β, IL-6, IL-10, TNFα, STNFR1, STNFR2, and Adiponectin
Rudolph et al. (52)	2008	Chemokines Are Associated with Delirium After Cardiac Surgery	Observational study	PO cardiac surgery	24	Good	Confusion assessment method-ICU	Mini-mental state examination	IL-1β, IL-1β, IL-6, IL-8, IL-10, TNFα, STNFR2, IFNβ, IP-10, MIP-1, MIG, EOTAXIN, RANTES, CCL-2, GM-CSF
Saller et al. (53)	2019	A case series on the value of tau and neurofilament protein levels to predict and detect delirium in cardiac surgery patients	Observational study	PO cardiac surgery	9	Poor	Confusion assessment method-ICU	Mini-mental state examination	NfL GFAP, tau
Simons et al. (54)	2018	Temporal biomarker profiles and their association with ICU acquired delirium: A cohort study	Observational study	ICU patients	50	Fair	Confusion assessment method-ICU	Not assessed	IL-1β, IL-6, Tau, APOE, neopterin, TNFα
Slor et al. (55)	2019	The trajectory of C-reactive protein serum levels in older hip fracture patients with postoperative delirium	Observational study	PO orthopedic surgery	121	Good	Confusion assessment method-ICU	Mini-mental state examination	CRP
Tomasi et al. (56)	2015	Baseline acetylcholinesterase activity and serotonin plasma levels are not associated with delirium in critically ill patients	Observational study	ICU patients	77	Fair	Confusion assessment method-ICU	Not assessed	AchE, and Serotonin
van den Boogaard et al. (57)	2011	Biomarkers associated with delirium in critically ill patients and their relation with long-term subjective cognitive dysfunction; indications for different pathways governing delirium in inflamed and noninflamed patients	Observational study	ICU patients	100	Fair	Confusion assessment method-ICU	Dutch translation of the cognitive-failure questionnaire	S100B, IL-1β, IL-6, IL-8, IL-10, IL-17, IL-18, IL-1ra, A-b, MCP-1, MIF, HNP-1, CRP, PCT, Cortisol
Van Munster et al. (58)	2008	Time-course of cytokines during delirium in elderly patients with hip fractures	Observational study	PO orthopedic surgery	98	Fair	Confusion assessment method-ICU	Informant Questionnaire on Cognitive decline short form	IL-1β. IL-6, IL-8, IL-10, IL-12, TNFα
van Munster et al. (59)	2009	Markers of cerebral damage during delirium in elderly patients with hip fracture	Observational study	PO orthopedic surgery	120	Fair	Confusion assessment method-ICU	Informant Questionnaire on Cognitive decline short form	S100B, NSE
van Munster et al. (60)	2010	Cortisol, interleukins and S100B in delirium in the elderly	Observational study	PO orthopedic surgery	120	Fair	Confusion assessment method-ICU	Informant Questionnaire on Cognitive decline short form	Cortisol, IL-6, IL-8, S100B
van Munster et al. (61)	2010	Serum S100B in elderly patients with and without delirium	Observational study	General ward patients	412	Fair	Confusion assessment method-ICU	Informant Questionnaire on Cognitive decline short form	S100B
Zhang et al. (60)	2014	Prediction of delirium in critically ill patients with elevated C-reactive protein	Observational study	ICU patients	223	Fair	Confusion assessment method-ICU	Not assessed	CRP
Zhao et al. (62)	2019	Low Plasma Cholinesterase Activity is Associated with Postoperative Delirium After Noncardiac Surgery in Elderly Patients: A Prospective Observational Study	Observational study	PO orthopedic surgery	206	Fair	Confusion assessment method-ICU	Mini-mental state examination	AChE, BuChE
Zhu et al. (63)	2017	Serum galectin-3 levels and delirium among postpartum intensive care unit women	Observational study	ICU patients	412	Fair	Confusion assessment method-ICU	Not assessed	Galectin-3, S100B, CRP

Sixty-seven biomarkers were investigated within the 55 studies selected (Supplementary Table S2). Twenty-two studies investigated the association of IL-6 and delirium, which made it the most investigated biomarker. The second-most investigated biomarker was C-reactive protein (CRP) with 18 studies. S100β was the third-most investigated biomarker, with 12 studies, followed by cortisol, with 10 studies.

Ten biomarkers were investigated in four or more studies and were included in the meta-analysis. Analyses of NSE, TNFα, IL-1β, IL-8, and IL-10 serum concentrations at hospital admission showed no statistically significant differences when comparing patients who did and did not develop delirium during hospitalization. These are reported in the Supplemental material. Analyses of the other five biomarkers [cortisol, CRP, IL-6, S100 β and acetylcholinesterase (AchE)] are summarized below.

Twenty-two studies conducted multi-regression analyses to investigate the possible independence of the variable of interest (Table 2). The most common confounding variables included in the multi-regression analyses were age, sex, Acute Physiology and Chronic Health Evaluation II (APACHE II) score, intubation, living alone, physical restraint, alcohol consumption, smoking, type of medical condition, hospital length of stay before ICU admission, American Society of Anesthesiologists Preoperative score (ASA score), type of surgery, sequential organ failure assessment (SOFA) score, and the existence of cognitive impairment pre-hospitalization. The most common delirium assessment tool used in the studies was the confusion assessment method-intensive care unit (CAM-ICU). Fifty-one out of fifty-five studies used CAM-ICU to assess delirium (Table 1). The most common cognitive test used to assess cognitive impairment was the Mini-Mental State Examination with 15 studies, followed by the Informant Questionnaire on Cognitive Decline with seven studies. Cognition was assessed as part of the exclusion criteria before study enrollment in 16 studies, excluding patients who had lower cognitive scores. In six studies, a cognitive test was included to investigate whether lower cognitive scores would be associated with a greater risk of delirium.

**Table 2:** Table showing papers that investigated biomarkers for delirium and controlled for illness.

**Source**	**No. of patients**	**Serum concentration mean (SD)**		**Patient type or setting**
		**Delirium**	**No delirium**	
**Cortisol (ng/ml)**				
Plaschke et al. (48)	114	11.0 (0.5)	8.6 (0.4)	Post-cardiac surgery
van Munster et al. (60)	120	22.0 (2.9)	20.6 (1.1)	Critically ill
Van den Boogaard et al. (57)	100	20.4 (4.4)	14.0 (3.7)	Critically ill
Cerejeira et al. (20)	101	16.2 (7.5)	18.6 (8.7)	Post-cardiac surgery
Kazmieski et al. (32)	113	14.1 (5.9)	12.6 (5.0)	Post-cardiac surgery
Colkesen et al. (22)	52	13.0 (10.0)	5.0 (5.0)	Post-cardiac surgery
Nguyen et al. (64)	128	14.4 (2.1)	8.4 (2.0)	Critically ill
Ma et al. (40)	119	12.7 (5.3)	8.6 (3.3)	Post-cardiac surgery
**C-Reactive Protein (mg/L)**				
Belooseky et al. (17)	38	246.0 (120.0)	128.0 (50.0)	Post-orthopedic surgery
van den Boogaard et al. (57)	100	56.0 (24.0)	47.0 (15.0)	Critically ill
McGrane et al. (41)	87	281.0 (100.0)	107.0 (61.0)	Critically ill
Cerejeira et al. (20)	101	117.0 (38.0)	91.0 (51.0)	Post-cardiac surgery
Girard et al. (10)	138	123.3 (40.0)	93.0 (40.0)	Critically ill
Osse et al. (46)	125	33.0 (30.0)	28.0 (20.0)	Post-cardiac surgery
Ritchie et al. (50)	710	43.4 (20.0)	16.1 (14.0)	General ward
Zhang et al. (65)	223	120.5 (30.0)	57.5 (40.0)	Critically ill
Cereghetti et al. (19)	412	254.0 (113.0)	166.0 (114.0)	Post-cardiac surgery
Zhu et al. (63)	618	236.0 (46.0)	186.0 (48.0)	Critically ill
Erikson et al. (25)	121	40.0 (10.0)	30.0 (10.0)	Critically ill
Knaak et al. (35)	22	280.0 (120.0)	180.0 (60.0)	Post-surgical (general)
Slor et al. (55)	314	155.0 (130.0)	45.0 (35.0)	Post-orthopedic surgery
Lu et al. (38)	152	31.7 (4.3)	26.5 (5.2)	Critically ill (breast cancer)
Ren et al. (49)	206	86.6 (62.0)	37.8 (32.3)	Post-orthopedic surgery
**IL-6 (pg/ml)**				
Belooseky et al. (17)	38	47.6 (11.0)	35.9 (5.4)	Post-orthopedic surgery
Rudolph et al. (52)	24	295.8 (222.0)	144.1 (179.0)	Post-cardiac surgery
Van Munster et al. (58)	98	59.0 (21.0)	36.0 (16.0)	Post-orthopedic surgery
Plaschke et al. (48)	114	280.0 (120.0)	220.0 (80.0)	Post-cardiac surgery
van Munster et al. (60)	120	78.0 (48.0)	36.0 (11.0)	Critically ill
van den Boogaard et al. (57)	100	61.0 (52.0)	52.0 (29.0)	Critically ill
Cerejeira et al. (20)	101	85.9 (6.6)	84.9 (1.0)	Post-cardiac surgery
Liu et al. (37)	338	6.6 (5.9)	3.6 (3.0)	Post-orthopedic surgery
Alexander et al. (14)	53	440.0 (100.0)	180.0 (110.0)	Critically ill
Ritter et al. (51)	78	225.0 (117.0)	145.0 (140.0)	Critically ill
Jorge-Ripper et al. (31)	144	24.8 (4.3)	2.9 (1.3)	General ward
Simons et al. (54)	50	325.0 (140.0)	600.0 (471.0)	Critically ill
Chen et al. (21)	22	19.7 (11.0)	9.9 (7.1)	Post-cardiac surgery
Erikson et al. (25)	121	305.0 (90.0)	160.0 (70.0)	Critically ill
McNeil et al. (8)	156	124.0 (38.0)	81.0 (60.0)	General ward
Peng et al. (47)	272	17.9 (7.1)	15.7 (6.6)	Post-orthopedic surgery
Lv et al. (39)	221	114.0 (66.0)	56.0 (26.0)	Post-cardiac surgery
**S100B (ng/mL)**				
Linstedt et al. (66)	117	0.24 (0.23)	0.14 (0.03)	General ward
van Munster et al. (59)	120	0.16 (0.10)	0.10 (0.10)	Post-orthopedic surgery
van Munster et al. (61)	120	0.16 (0.06)	0.12 (0.03)	Critically ill
Grandi et al. (28)	60	0.83 (0.40)	0.88 (0.40)	Critically ill
Nguyen et al. (64)	128	0.13 (0.01)	0.09 (0.01)	Critically ill
Jorge-Ripper et al. (31)	144	0.16 (0.05)	0.03 (0.03)	General ward
Erikson et al. (25)	121	0.30 (0.10)	0.15 (0.05)	Critically ill
Li et al. (67)	186	0.07 (0.04)	0.03 (0.01)	Post-orthopedic surgery
**Acetylcholinesterase (U/mL)**				
Cerejeira et al. (20)	101	2.4 (0.6)	2.7 (0.5)	Post-cardiac surgery
Tomasi et al. (56)	77	1.5 (1.6)	1.7 (1.2)	Critically ill
Jackson et al. (29)	110	2.7 (1.7)	2.0 (1.3)	General ward
Muller et al. (44)	650	4.4 (1.0)	4.2 (0.8)	Post-surgical (general)
Zhao et al. (62)	206	4.3 (1.8)	9.8 (2.5)	Post-surgical (general)
Ma et al. (40)	119	1.2 (0.6)	2.0 (1.3)	Post-cardiac surgery
Adam et al. (12)	114	4.2 (0.6)	4.5 (0.6)	Post-surgical (general)

### Higher IL-6 serum concentration at hospital admission is associated with delirium

**Figure 1:**
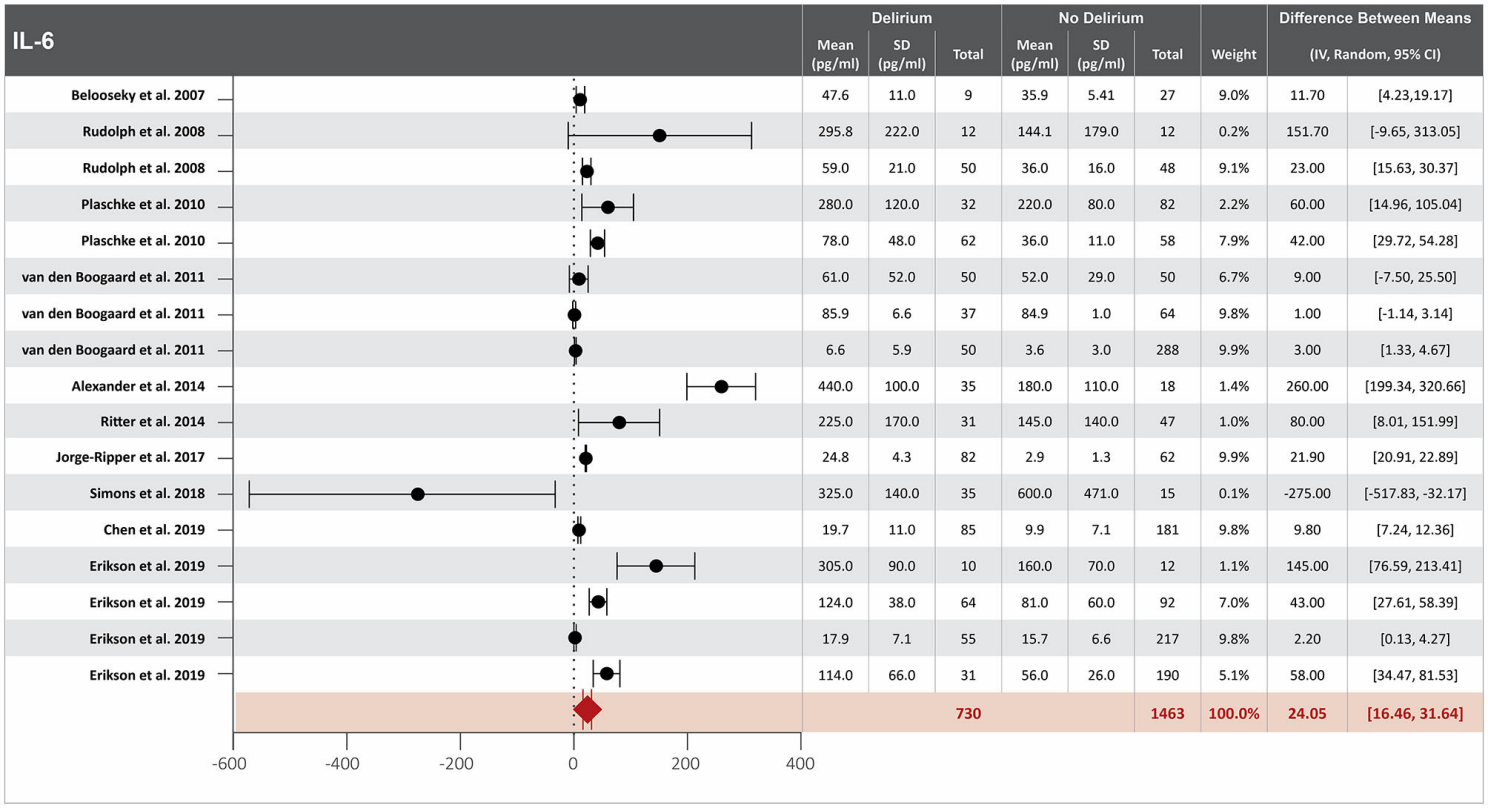
Forest plot showing the difference in mean serum concentration of interleukin-6 (IL-6) at hospital admission between patients who did and did not develop delirium during hospitalization. The difference between the means was 24.05 pg/ml and the heterogeneity between the studies with a Tau^2^ of 151.47, Chi^2^ of 832.4, a df of 16 with a *p* < 0.00001, and I^2^ 98%, Z of 6.21 with a *p* < 0.00001 for total overall effect.

Twenty-two studies investigated the association between IL-6 serum concentration at hospital admission and delirium during hospitalization (8, 13, 14, 16–18, 20, 21, 23, 25, 31, 37, 39, 42, 47, 48, 51, 52, 54, 57, 58, 60). Mean IL-6 serum concentrations at hospital admission were reported in 17 studies, with a difference between the means of 24.05 pg/ml greater in patients who developed delirium than in those who did not (8, 14, 17, 20, 21, 23, 25, 31, 37, 39, 47, 48, 51, 54, 57, 58, 60). High heterogeneity was observed between these 17 studies, with a *p*-value of < 0.00001 for chi-square, I^2^ of 98%, and *p* < 0.00001 for the total overall effect (Figure 1). The standard mean difference between patients who developed delirium and those who did not was 1.18 pg/ml, with a 95% CI [0.73, 1.63] with a *p*-value of < 0.0001 for chi-square, I^2^ of 95%, and *p* < 0.0001 for the total overall effect. A subgroup analysis identified that 5 of the 22 studies found IL-6 serum concentration at hospital admission to be an independent variable for an increased likelihood of developing delirium, with a difference between the means of 29.66 pg/ml greater in patients who developed delirium than in those who did not (25, 34, 57, 60, 67). High heterogeneity was also observed between studies reporting mean serum concentration of IL-6 that controlled for illness, with p < 0.00001 for chi-square, I^2^ of 94%, and *p* < 0.0006 for the total overall effect. A subgroup analysis that included exclusively post-surgical patients still showed high heterogeneity between the studies and a mean difference in serum concentration of IL-6 at hospital admission between patients who developed delirium and those who did not was 29.04 pg/ml, *p* = 0.0001 for chi-square, I^2^ of 82%, and *p* = 0.0002 for the total overall effect (Supplementary Figure S1) (17, 48, 52, 58, 60).

### Higher CRP serum concentration at hospital admission is associated with delirium

**Figure 2:**
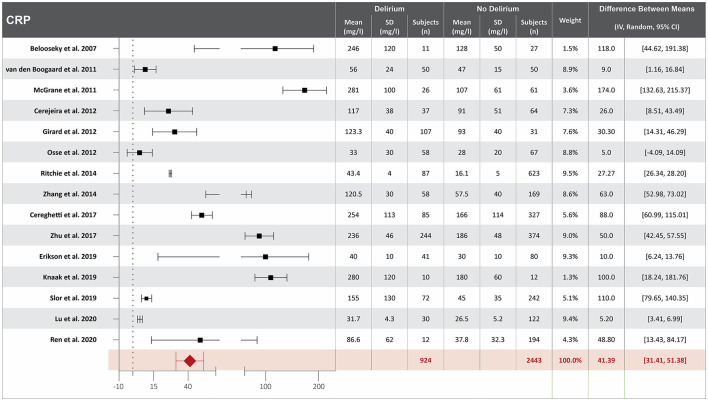
Forest plot showing the difference in mean serum concentration of C-reactive protein (CRP) at hospital admission between patients who did and did not develop delirium during hospitalization. The difference between the means was 41.39/mg/l and the heterogeneity between the studies with a Tau^2^ of 273.96, Chi^2^ of 759.6, a df of 14 with a *p* < 0.00001, and I^2^ 98%, Z of 8.13 with a *p* < 0.00001 for total overall effect.

Eighteen studies investigated the association between CRP serum concentration at hospital admission and delirium during hospitalization (10, 13, 17, 19, 20, 23, 25, 35, 38, 41, 46, 47, 49, 50, 55, 57, 63, 65). Mean CRP serum concentrations at hospital admission were reported in 15 studies, with a difference of 41.39 mg/L between the means that is greater in patients who developed delirium than in those who did not (10, 13, 17, 19, 20, 23, 25, 35, 38, 41, 49, 50, 55, 57, 65). High heterogeneity was observed between these 15 studies, with a *p*-value of < 0.00001 for chi-square, I^2^ of 98%, and *p* < 0.00001 for the total overall effect (Figure 2). The standard mean difference between patients who developed delirium and those who did not was 1.58 mg/L, with a 95% CI [0.79, 2.37] with a *p*-value of < 0.0001 for chi-square, I^2^ of 98%, and *p* < 0.0001 for the total overall effect. A subgroup analysis identified that nine of the eighteen studies found CRP serum concentration at hospital admission to be an independent variable for increased likelihood of delirium, with a difference of 46.65 mg/L between the means that is greater in patients who developed delirium than in those who did not (10, 17, 19, 20, 35, 41, 49, 50, 65). High heterogeneity was also observed between studies reporting mean CRP serum concentration that controlled for illness, with a *p*-value of < 0.00001 for chi-square, I^2^ of 98%, and a *p*-value of < 0.00001 for the total overall effect. A subgroup analysis that included exclusively post-surgical patients showed moderate heterogeneity between the studies and a mean difference in serum concentration of CRP at hospital admission between patients who developed delirium and those who did not was 11.93 mg/L, with a *p*-value of 0.03 for chi-square, I^2^ of 67%, and a *p*-value of 0.0006 for the total overall effect (Supplementary Figure S2) (20, 46, 47, 49, 55).

### Higher S100β serum concentration at hospital admission is associated with delirium

**Figure 3:**
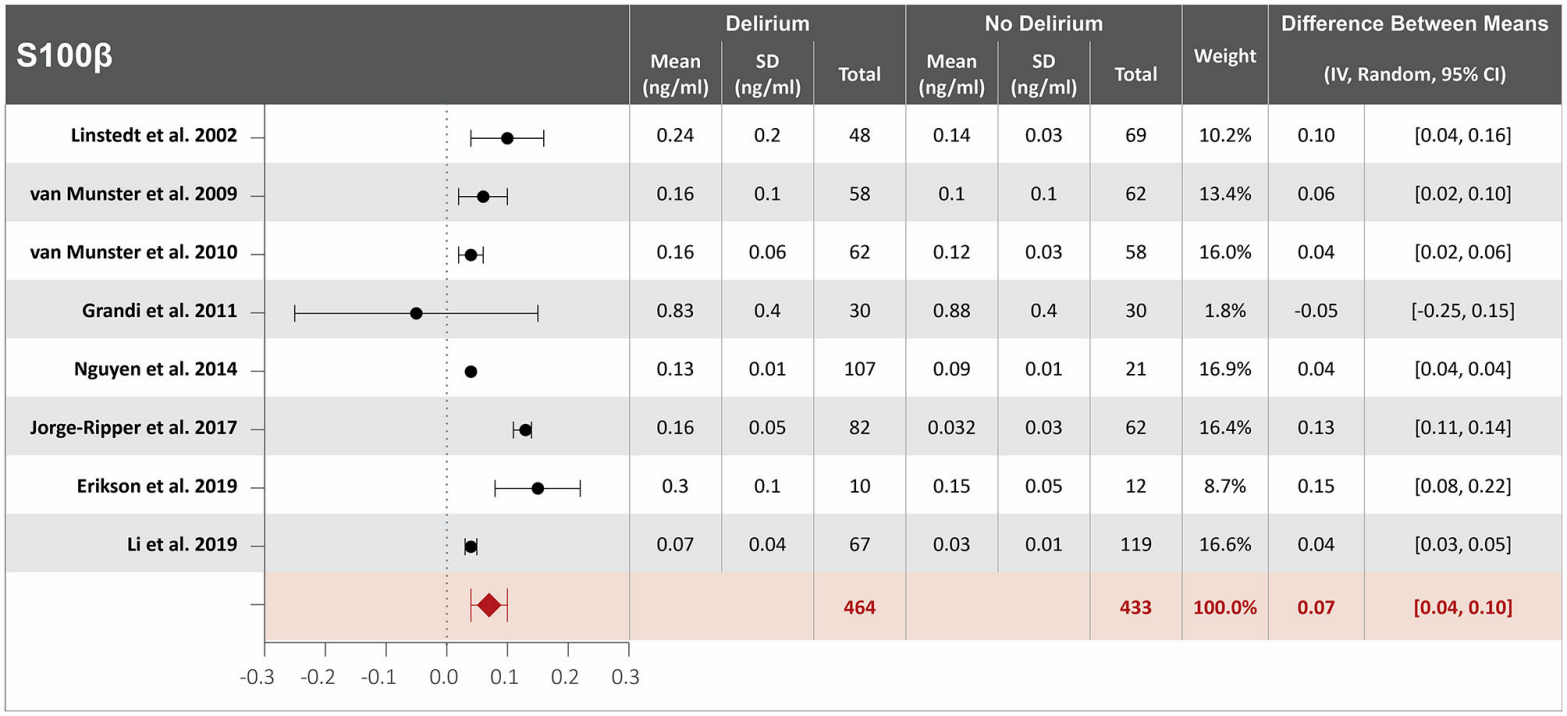
Forest plot showing the difference in mean serum concentration of calcium-binding protein S-100β at hospital admission between patients who did and did not develop delirium during hospitalization. The difference between the means was 0.07 ng/ml and the heterogeneity between the studies with a Tau^2^ of 0.00, Chi^2^ of 169.99, a df of 7 with a *p* < 0.00001, and I^2^ 96%, Z of 4.84 with a *p* < 0.00001 for total overall effect.

Twelve studies investigated the association between S100β serum concentration at hospital admission and delirium during hospitalization (8, 21, 25, 28, 31, 34, 36, 59, 61, 63, 64, 66). Mean S100 **β** serum concentrations at hospital admission were reported in eight studies, with a difference of 0.07 ng/ml between the means that is greater in patients who developed delirium than in those who did not. High heterogeneity was observed between these eight studies, with a *p*-value of < 0.00001 for chi-square, I^2^ of 96%, and a *p*-value of < 0.00001 for the total overall effect (Figure 3) (25, 28, 31, 36, 59, 61, 64, 66). The standard mean difference between patients who developed delirium and those who did not was 1.30 ng/ml, with a 95% CI [0.70, 1.89] with a *p*-value of < 0.0001 for chi-square, I^2^ of 95%, and a *p*-value of < 0.0001 for the total overall effect. A subgroup analysis identified that eight of the twelve studies found S100β serum concentration at hospital admission to be an independent variable for increased likelihood of delirium, with a difference between the means of 0.05 ng/ml greater in patients who developed delirium than in those who did not (25, 28, 31, 36, 59, 61, 64, 66). High heterogeneity was also observed between studies reporting mean S100 β serum concentration that controlled for illness, with a *p*-value of < 0.00001 for chi-square, I^2^ of 97%, and a *p*-value of < 0.0001 for the total overall effect. A subgroup analysis that included exclusively post-surgical patients showed no heterogeneity between the studies and a mean difference in serum concentration of S100β at hospital admission between patients who developed delirium and those who did not was 0.04 ng/ml, a *p*-value of 0.76 for chi-square, I^2^ of 0%, and a *p*-value of < 0.00001 for the total overall effect (Supplementary Figure S3) (36, 59, 61).

### Higher cortisol serum concentration at hospital admission is associated with delirium

**Figure 4:**
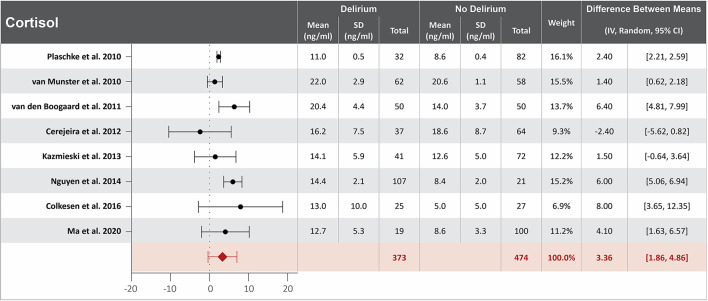
Forest plot showing the difference in mean serum concentration of cortisol at hospital admission between patients who did and did not develop delirium during hospitalization. The difference between the means was 3.36 ng/ml and the heterogeneity between the studies with a Tau^2^ of 3.62, Chi^2^ of 102.02, a df of 7 with a *p* < 0.00001, and I^2^ 93%, Z of 4.39 with a *p* < 0.00001 for total overall effect.

Ten studies investigated the association between cortisol serum concentration at hospital admission and delirium during hospitalization (20, 22, 32, 33, 40, 43, 48, 57, 60, 64). Mean cortisol serum concentrations at hospital admission were reported in eight studies, with a difference of 3.36 ng/ml between the means that is greater in patients who developed delirium than in those who did not (20, 22, 32, 40, 48, 57, 60, 64). High heterogeneity was observed between studies that reported mean serum concentration of cortisol, with a *p*-value of < 0.00001 for chi-square, I^2^ of 93%, and a *p*-value of < 0.0001 for the total overall effect (Figure 4). The standard means difference between patients who developed delirium and those who did not was 1.55 ng/ml, with a 95% CI [0.62, 2.48] with a *p*-value of < 0.0001 for chi-square, I^2^ of 97%, and a *p*-value of *p* = 0.001 for the total overall effect. A subgroup analysis identified that four of the ten studies found cortisol serum concentration at hospital admission to be an independent variable for increased likelihood of delirium, with a difference of 3.32 ng/ml between the means that is greater in patients who developed delirium than in those who did not (22, 40, 43, 48). High heterogeneity was also observed between studies reporting mean cortisol serum concentration that controlled for illness, with a *p*-value of < 0.00001 for chi-square, I^2^ of 91%, and a *p*-value of < 0.0001 for the total overall effect. A subgroup analysis that included exclusively post-surgical patients showed moderate heterogeneity between the studies and a mean difference in serum concentration of cortisol at hospital admission between patients who developed delirium and those who did not was 2.02 ng/ml, a *p*-value of 0.12 for chi-square, I^2^ of 52%, and a *p*-value of 0.006 for the total overall effect (Supplementary Figure S4) (32, 40, 60).

### Lower acetylcholinesterase serum concentration at hospital admission might be associated with delirium

**Figure 5:**
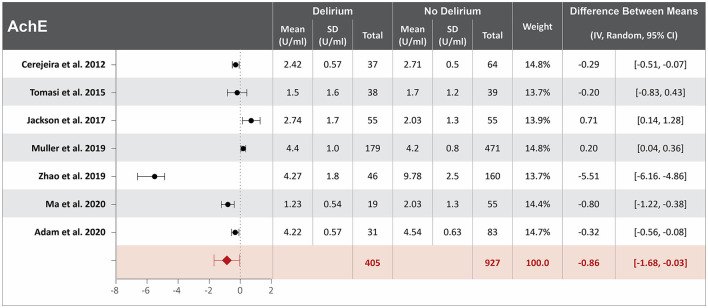
Forest plot showing the difference in mean serum concentration of acetylcholinesterase at hospital admission between patients who did and did not develop delirium during hospitalization. The difference between the means was −0.86 U/ml and the heterogeneity between the studies with a Tau^2^ of 1.19, Chi^2^ of 299.7, a df of 6 with a *p* < 0.00001, and I^2^ 98%, Z of 2.03 with a *p* = 0.04 for total overall effect.

Seven studies investigated the association between acetylcholinesterase (AchE) serum concentration at hospital admission and delirium during hospitalization (12, 20, 29, 40, 44, 56, 62). Mean AchE serum concentrations at hospital admission were reported in all seven studies, with a difference of 0.86 U/ml between the means that is lower in patients who developed delirium than in those who did not. High heterogeneity was observed between these seven studies, with a *p*-value of < 0.0001 for chi-square, I^2^ of 98%, and a *p*-value of 0.04 for the total overall effect (Figure 5) (12, 20, 29, 40, 44, 56, 62). The standard means difference between patients who developed delirium and those who did not was −0.50 U/ml, with a 95% CI [−1.18, 0.19], a *p*-value of < 0.0001 for chi-square, I^2^ of 96%, and a *p*-value of 0.16 for the total overall effect. A subgroup analysis identified that three of the seven studies found AchE serum concentration at hospital admission to be an independent variable for identifying those patients with an increased likelihood of developing delirium, with a difference of 1.4 U/ml between the means, which was lower in patients who developed delirium than in those who did not (12, 20, 40). High heterogeneity was also observed between studies reporting mean AchE serum concentration that controlled for illness, with a *p*-value of < 0.00001 for chi-square, I^2^ of 98%, and a *p*-value of < 0.02 for the total overall effect. A subgroup analysis that included exclusively post-surgical patients still showed high heterogeneity between the studies and a mean difference in serum concentration of cortisol at hospital admission between patients who developed delirium and those who did not was −0.26. U/ml, a *p*-value of < 0.000 1 for chi-square, I^2^ of 99%, and a *p*-value of < 0.0001 for the total overall effect (Supplementary Figure S5) (12, 20, 40, 44, 62).

## Discussion

Our meta-analysis found evidence that serum concentrations, at hospital admission, of three inflammatory proteins (cortisol, CRP, and IL-6) and one biomarker for blood-brain barrier leakage (S100β) were higher in patients who subsequently developed delirium during hospitalization in comparison to those who did not. Moreover, our systematic review showed that lower serum concentration of acetylcholinesterase at hospital admission was possibly associated with an increased likelihood of developing delirium during hospitalization.

### Systemic inflammation, increased blood-brain barrier permeability, and delirium

Delirium is a complex syndrome that may have multiple triggering factors (1, 8, 9). It is an enormous challenge to isolate variables that can trigger delirium. It has been demonstrated that delirium is associated with systemic inflammation, neuroinflammation, and consequently, increased BBB permeability (1, 8, 9, 68, 69). Preclinical experiments have shown that systemic inflammation, characterized by an increased serum concentration of IL-6, induces neuroinflammation and BBB breakdown (45, 70, 71). Moreover, neuroinflammation has been demonstrated to lead to neurotransmitter imbalance and possibly cognitive dysfunction (72). For instance, dopamine overexpression is associated with microglia priming and neuronal apoptosis, which are associated with working-memory dysfunction (72).

Many factors can trigger neuroinflammation, and systemic inflammation is one of those factors (72). C-reactive protein is considered a systemic inflammatory biomarker that has been linked to increased BBB permeability (65). Our systematic review identified nine studies that showed elevated CRP serum concentrations at hospital admission as a predictor of delirium. For example, Zhang et al. (65) investigated 223 ICU patients, showing that those who developed delirium had higher CRP serum concentration compared to those who did not develop delirium during hospitalization (120.5 vs. 57.5 mg/L) (65). Moreover, the same study showed that after adjusting for confounding variables, including age, sex, Acute Physiology and Chronic Health Evaluation II (APACHE II) score, intubation, living alone, physical restraint, alcohol consumption, smoking, type of medical condition, and hospital length of stay before ICU admission, in a logistic regression model, elevated CRP remained an independent predictor of delirium (odds ratio, 1.07; 95% confidence interval, 1.01–1.15) (65). Our systematic review identified 17 studies that showed elevated IL-6 serum concentrations as a predictor of delirium. For instance, Erikson et al. (65) found that patients who developed delirium during hospitalization had greater IL-6 serum concentration compared to those who did not, 138.3 pg/ml (28.0–297.7) vs. 53.6 pg/ml (109.3–505.0), respectively (65). Thus, there is a strong signal that elevated serum concentration of inflammatory markers may be a predisposing factor to increase patient vulnerability to developing delirium during hospitalization. This is supported by greater serum concentrations of CRP and IL-6 in patients who developed delirium during hospitalization (65, 73, 74).

Preclinical and clinical studies have shown that increased BBB permeability is associated with greater S100β serum concentration (75). Increased BBB permeability facilitates the entry of inflammatory proteins into the central nervous system, promoting neuroinflammation (75). It is reasonable to argue that the brains of patients who have increased BBB permeability are more vulnerable to the effects of systemic inflammation when compared to patients with intact BBBs. For these vulnerable patients, events that normally would not trigger neuroinflammation, and consequently cognitive dysfunction, might initiate neuroinflammation resulting in cognitive dysfunction due to this increased BBB permeability. Erikson et al. (65) showed a positive, linear, and moderate correlation between S100β and IL-6 serum concentrations (*r* = 0.489, *p* = 0.021) which supports the argument of a link between systemic inflammation and BBB permeability (65). Our systematic review identified 12 studies that investigated S100β serum concentration as a predictor for delirium during hospitalization, eight of which controlled for illness, and six out of these eight controlled for cognitive impairment before hospitalization (8, 21, 25, 28, 31, 34, 36, 59, 61, 63, 64, 66). For example, van Munster et al. (60) investigated 120 patients, showing that S100β serum concentration was an independent variable for predicting delirium after a multi-regression analysis, controlling for illness, and even including pre-existing cognitive impairment (60). Moreover, a subgroup analysis identified that surgical patients who developed delirium during hospitalization had a mean difference in S100β serum concentration at hospital admission of 0.04 ng/ml greater than surgical patients who did not develop delirium during hospitalization.

### Hypothalamic-pituitary axis dysfunction and delirium

Hypothalamic-pituitary axis (HPA) dysfunction is another factor that has been proposed to increase patients' vulnerability to developing delirium during hospitalization (1). Persistent high serum concentration of cortisol has been proposed as indicators of HPA dysfunction, both of which make patients more vulnerable to systemic inflammation due to the inability of the body to properly respond to inflammation (1). Moreover, HPA dysfunction has been shown to be associated with changes in the brain's neurophysiology and consequently in the homeostasis of dopamine (1). Our systematic review showed that higher serum concentrations of cortisol and lower serum concentrations of acetylcholinesterase at hospital admission were each associated with a greater likelihood of developing delirium during hospitalization. For instance, Ma et al. (40) investigated 119 patients, showing that after isolating patient illness severity, patients who developed delirium during hospitalization had higher serum concentrations of cortisol and lower serum concentrations of acetylcholinesterase compared to patients who did not develop delirium during hospitalization (40).

### Potential etiologies of delirium

Greater serum concentrations of IL-6, CRP, and cortisol can be used as markers of systemic inflammation (22, 32, 33, 43, 60, 64, 76). Chronic inflammation has been linked to BBB dysfunction (70, 71, 75, 77). It is unknown whether greater S100β serum concentration could be a result of chronic inflammation, increasing patients' vulnerability to develop delirium, or greater S100β serum concentration might be the cause for increased vulnerability to delirium (24, 46, 54). It is also unknown whether chronic inflammation could also modulate acetylcholinesterase activity.

Studies have shown an association between age, chronic use of anticholinergic drugs, and lower serum concentration of acetylcholinesterase (1, 78, 79). The cholinergic system is vital to modulate the neurotransmitter balance in the brain (78). Thus, it has been argued that an impaired cholinergic system, leading to neurotransmitter unbalance might increase the patient's vulnerability to developing delirium. It has been shown that the inhibition of the postsynaptic cholinergic receptors is associated with delirium-like symptoms such as confusion, acute cognitive impairment, and lack of attention (1). Another reason for a decline in acetylcholinergic receptors concentration is aging, which also could result in a reduction of the acetylcholinesterase activity leading to greater vulnerability to developing delirium in this population (1, 78, 79). It is important to highlight that studies that control for age and cognitive impairment pre-hospitalization have shown an association between lower serum concentration of acetylcholinesterase and greater risk for delirium during hospitalization (1, 29, 40, 56). However, these studies have not controlled for the use of anticholinergic medications pre-hospitalization. Combining all the data together, it can be hypothesized that greater vulnerability to developing delirium during hospitalization might be an effect of chronic inflammation, lower acetylcholinergic activity, and BBB dysfunction, and all of these factors might be potentiated by aging.

### Gaps in the research literature

More studies should focus on neuroinflammation and delirium, investigating neuroinflammation as a potentiator of delirium. For example, in our search only three studies investigated the association of biomarkers for microglia priming (neopterin) and delirium, indicating that more studies focusing on microglia-priming markers are needed (24, 46, 54).

Moreover, more studies focusing on the pathophysiology of delirium to identify any common pathways to developing delirium during hospitalization should also be conducted. For instance, studies should investigate chronic inflammation and possible modulation of acetylcholinesterase activity. Finally, a prospective study that tests the predictive ability of a test panel comprised of the identified biomarkers and their respective thresholds to predict delirium would be truly important for this research field.

## Limitations

Our research has some limitations. First, most of the studies selected by our systematic review showed high heterogeneity, demonstrating high variability in any signals indicating that inflammatory markers, BBB permeability, HPA dysfunction, and cholinergic burden at hospital admission might be associated with a greater likelihood of developing delirium during hospitalization. Another limitation is the high heterogenicity of the population analyzed since we have pooled critically ill patients, general-ward patients, and post-surgical procedure patients into the same analysis. In a highly variable population, the effect of inflammation and neurotransmitter imbalance pre-hospitalization could be an important confounder affecting the serum concentrations of the biomarkers analyzed. Despite that, our meta-analysis found an association between elevated mean serum concentrations of inflammatory markers and neurotransmitter imbalances at hospital admission and the development of delirium during hospitalization. The consistency of these results across a highly variable population increases the translatability of our findings because, in a real clinical scenario, patients have a high variability of diseases and conditions. More studies focusing on biomarkers for neurotransmitter imbalances before hospitalization and the likelihood of developing delirium during hospitalization are needed, such as but not limited to, the overexpression of dopamine. The findings of this systematic review and meta-analysis should be interpreted within the context of the included studies.

## Conclusion

Our meta-analysis indicates that greater serum concentrations of pro-inflammatory proteins (cortisol, C-reactive protein, and IL-6) at hospital admission are associated with a greater likelihood of patients developing delirium when compared to patients with lower serum concentrations of pro-inflammatory proteins at hospital admission, and this is independent of the severity of illness. Additionally, a greater serum concentration of S100β at hospital admission is associated with a greater likelihood to develop delirium during hospitalization. A lower serum concentration of acetylcholinesterase was also associated with an increased vulnerability to developing delirium during hospitalization. Our meta-analysis found a signal that patients with hypothalamic-pituitary axis dysfunction, increased blood-brain barrier permeability, and chronic overload of the cholinergic system, at hospital admission, are more vulnerable to developing delirium during hospitalization. Gaps in the literature were identified, such as the small number of studies investigating biomarkers for neurotransmitter imbalance and microglia priming pre-hospitalization.

## Data availability statement

The original contributions presented in the study are included in the article/Supplementary material, further inquiries can be directed to the corresponding author.

## Author contributions

TB was responsible for the hypothesis generation and conception of this study. TB and SR contributed to the study design and data interpretation. TB, ER, and SR were responsible for writing the article. TB, ER, and MN performed data acquisition and conducted data analysis. All authors have agreed with the final version of the manuscript before submission.
