# Supplementary material for: Meta-analysis of serological biomarkers at hospital admission for the likelihood of developing delirium during hospitalization
Source: Front Neurol. 2023 Jun 9;14:1179243. doi: 10.3389/fneur.2023.1179243 (PMC10288875; doi:10.3389/fneur.2023.1179243)
Supplement: Supplementary file 1 [file Data_Sheet_1.DOCX]

Table S1. Table showing search term(s)pre-established keyword(s) used for search

| **Search Sequence** | **Search Term(s)/Pre-established keyword(s)** |
| --- | --- |
| 1 | Delirium |
| 2 | Biomarkers |
| 3 | Cognitive dysfunction |
| 4 | Delirium/ |
| 5 | Neurocognitive injury |
| 6 | Neurocognitive complication |
| 7 | Neurocognitive harm |
| 8 | Risk markers |
| 9 | Disease markers |
| 10 | End-product markers |
| Limits applied | Abstract available  English language |

| **Number of biomarkers** | **Biomarker** | **Number of studies that investigate each biomarker** | **Type of biomarker** |
| --- | --- | --- | --- |
| 1 | A-b | 2 | Disease |
| 2 | AchE | 7 | Disease |
| 3 | Arginine | 1 | Disease |
| 4 | Adiponectin | 1 | Disease |
| 5 | APOE | 2 | Risk |
| 6 | Albumin ratio | 1 | Risk |
| 7 | Beta amyloid | 2 | Risk |
| 8 | BDNF | 3 | Disease |
| 9 | BuchE | 3 | Disease |
| 10 | CCL-2 | 1 | Disease |
| 11 | Cortisol | 10 | Disease |
| 12 | CRP | 17 | Disease |
| 13 | D-dimer | 1 | Disease |
| 14 | E-selectin | 2 | Disease |
| 15 | Eotaxin | 1 | Disease |
| 16 | Galectin-3 | 1 | Disease |
| 17 | GFAP | 3 | End of product |
| 18 | GM-CSF | 1 | Disease |
| 19 | glutamine | 1 | Disease |
| 20 | IL-1α | 1 | Disease |
| 21 | IL-1β | 11 | Disease |
| 22 | IL-2 | 3 | Disease |
| 23 | IL-6 | 23 | Disease |
| 24 | IL-8 | 12 | Disease |
| 25 | IL-10 | 12 | Disease |
| 26 | IL-12 | 2 | Disease |
| 27 | IL-17 | 2 | Disease |
| 28 | IL-18 | 2 | Disease |
| 29 | IP-10 | 1 | Disease |
| 30 | ICAM-1 | 2 | Disease |
| 31 | IL-18 | 1 | Disease |
| 32 | IGF-1 | 3 | Disease |
| 33 | IFNγ | 3 | Disease |
| 34 | HVA | 3 | Disease |
| 35 | HNP-1 | 1 | Disease |
| 36 | PAI-1 | 2 | Disease |
| 37 | PCT | 2 | Disease |
| 38 | PECAM | 1 | Disease |
| 39 | Phe/LNAA | 1 | Disease |
| 40 | Prolactin | 1 | Disease |
| 41 | putrescine | 1 | Disease |
| 42 | MMP-9 | 1 | Disease |
| 43 | MPO | 2 | Disease |
| 44 | MCP-1 | 2 | Disease |
| 45 | MIF | 1 | Disease |
| 46 | Melatonin | 1 | Disease |
| 47 | Neopterin | 4 | Disease |
| 48 | NGAL | 1 | Disease |
| 49 | NSE | 10 | End of product |
| 50 | NfL | 5 | End of product |
| 51 | RAGE | 1 | Disease |
| 52 | S100B | 19 | End of product |
| 53 | SAA | 1 | Disease |
| 54 | Serotonin | 1 | Disease |
| 55 | serum lactase | 1 | Disease |
| 56 | spermidine | 1 | Disease |
| 57 | Soluble fibrinogen‐like protein 2 (sFGL2) | 1 | Disease |
| 58 | STNFR1 | 1 | Disease |
| 59 | STNFR2 | 1 | Disease |
| 60 | tau | 4 | End of product |
| 61 | TNFα | 11 | Disease |
| 62 | Trp/LNAA | 1 | Disease |
| 63 | Tyr/LNAA | 1 | Disease |
| 64 | trypophan | 3 | Disease |
| 65 | UCH-L1 | 2 | End of product |
| 66 | VCAM | 1 | Disease |
| 67 | VWF | 1 | Disease |

.

Table S2. List of biomarkers investigated

Figure S1. Forest plot showing the result of a sub-analysis only including surgical papers that investigated IL-6 serum concentration and association with increased likelihood for delirium during hospitalization.

**
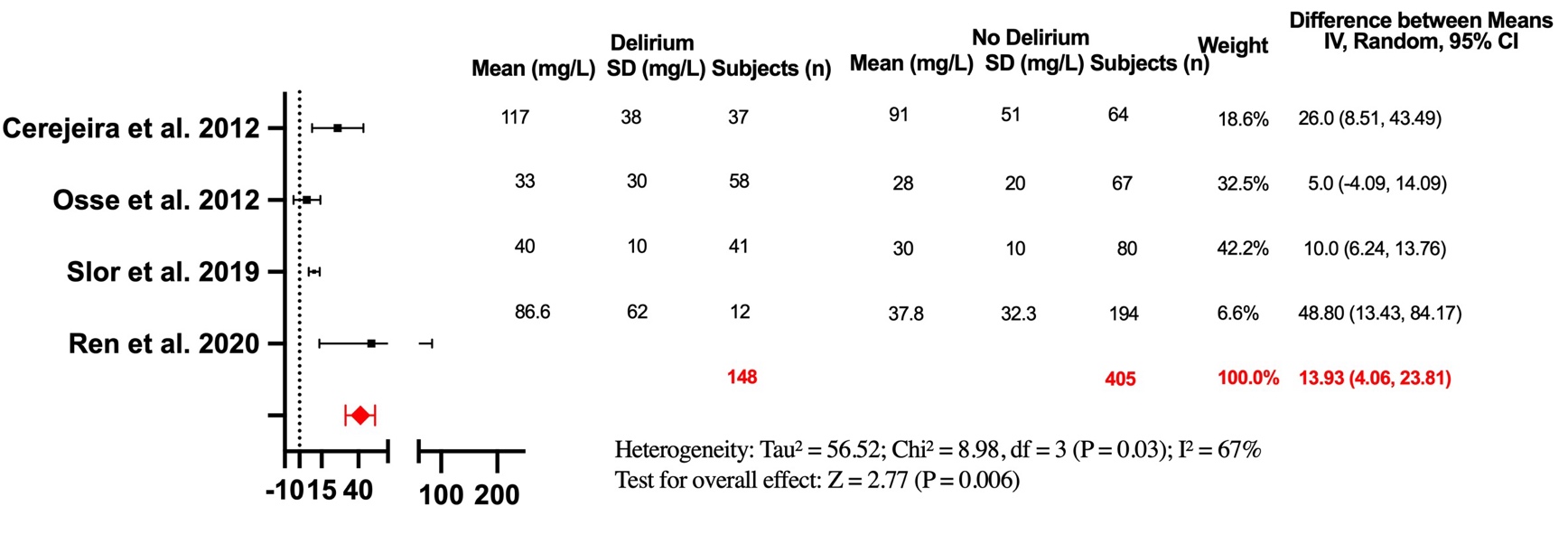
**

Figure S2. Forest plot showing the result of a sub-analysis only including surgical papers that investigated CRP serum concentration and association with increased likelihood for delirium during hospitalization.

Figure S3. Forest plot showing the result of a sub-analysis only including surgical papers that investigated S100β serum concentration and association with increased likelihood for delirium during hospitalization.

Figure S4. Forest plot showing the result of a sub-analysis only including surgical papers that investigated cortisol serum concentration and association with increased likelihood for delirium during hospitalization.

**
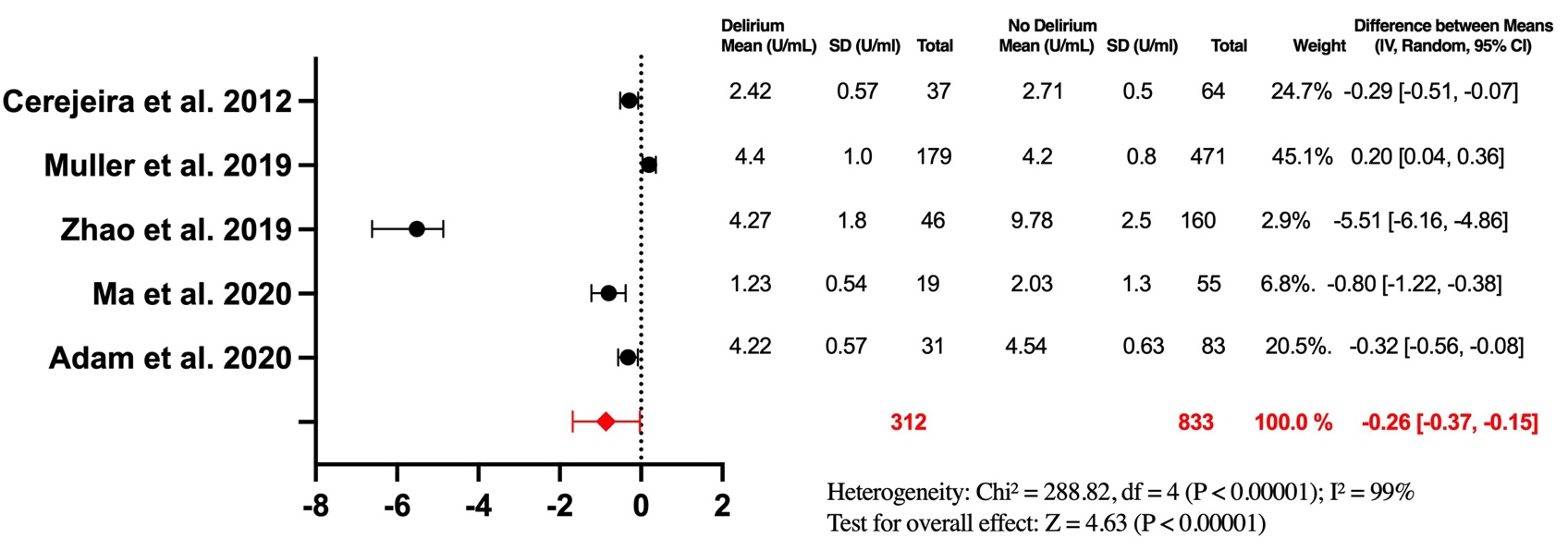
**

Figure S5. Forest plot showing the result of a sub-analysis only including surgical papers that investigated AchE serum concentration and association with increased likelihood for delirium during hospitalization.

**Higher NSE serum concentration at hospital admission is associated with delirium**

Eight papers investigated the association between NSE serum concentration at hospital admission and delirium. Seven out of nine papers found a significant difference between the means of NSE serum concentrations at hospital admission in patients who developed delirium in comparison to patients who did not develop delirium during hospitalization. Patients who developed delirium during hospitalization had higher baseline serum concentrations when compared to patients who did not develop delirium during hospitalization. The mean of the NSE serum concentration at hospital admission was reported in six papers. The difference between means in NSE serum concentrations at hospital admission between patients who developed and did not develop delirium was 2.49 ng/ml. High heterogeneity between the papers was observed with p<0.00001 for Chi-square, and an I^2^ of 100%. The p-value found for the total overall effect was 0.0002.

**Higher IL-1β at hospital admission is not associated with delirium**

Eleven papers investigated the association between IL-1b serum concentration at hospital admission and delirium. Six out of these eleven papers found a significant difference between the means of IL-1b serum concentration at hospital admission in patients who developed delirium compared to patients who did not develop delirium during hospitalization. Patients who developed delirium during hospitalization had higher baseline serum concentrations when compared to patients who did not develop delirium during hospitalization. The mean of the IL-1b serum concentration at hospital admission was reported in six papers. The difference between means in IL-1b serum concentrations at hospital admission between patients who developed and did not develop delirium was 0.44 pg/ml. Moderate heterogeneity between the papers was observed with p=0.01 for Chi-square, and an I^2^ of 66%. The p-value found for the total overall effect was 0.15.

**Higher IL-8 serum concentration at hospital admission is not associated with delirium**

Twelve papers investigated the association between IL-8 serum concentration at hospital admission and delirium. Seven out of these twelve papers found a significant difference between the means of IL-8 serum concentrations at hospital admission in patients who developed delirium in comparison to patients who did not develop delirium during hospitalization. Patients who developed delirium during hospitalization had higher baseline serum concentrations when compared to patients who did not develop delirium during hospitalization. The mean of the IL-8 serum concentration at hospital admission was reported in seven papers. The difference between means in IL-8 serum concentration at hospital admission between patients who developed delirium/POCD and did not develop delirium was 3.79 pg/ml. High heterogeneity between the papers was observed with p<0.00001 for Chi-square, and an I^2^ of 95%. The p-value found for the total overall effect was 0.54.

**Lower IL-10 serum concentration at hospital admission is not associated with delirium**

Thirteen papers investigated the association between IL-10 serum concentration at hospital admission and delirium. Only three out of these thirteen papers found a significant difference between the means of IL-10 serum concentration at hospital admission in patients who developed delirium in comparison to patients who did not develop delirium during hospitalization. Patients who developed delirium during hospitalization had lower baseline serum concentrations when compared to patients who did not develop delirium during hospitalization. The mean of the IL-10 serum concentration at hospital admission was reported in six papers. The difference between means in IL-10 serum concentration at hospital admission between patients who develop delirium and do not develop delirium/POCD was 3.33 pg/ml. High heterogeneity between the papers was observed with p<0.00001 for Chi-square, and an I^2^ of 91%. The p-value found for the total overall effect was 0.49.

**Higher TNF-a at hospital admission is associated with delirium**

Six papers investigated the association between TNF-α serum concentration at hospital admission and delirium. The mean TNF- α serum concentrations at hospital admission were reported in six papers with a difference between the means of 3.99 ng/mL greater in patients who developed delirium compared to patients who did not develop delirium. High heterogeneity was observed between these six papers with a p<0.00001 for Chi-square, I^2^ of 94%, and a p<0.00001 for the total overall effect. Three out of the six papers that investigated an association between TNF- α serum concentration at hospital admission and delirium conducted a multi-regression analysis showing no association between the difference in the means in TNF-α serum concentrations at hospital admission as an independent variable to an increased likelihood for delirium.
